# Stature estimation for Saudi men based on different combinations of upper limb part dimensions

**DOI:** 10.1097/MD.0000000000025840

**Published:** 2021-05-14

**Authors:** Altayeb Abdalla Ahmed

**Affiliations:** a Department of Basic Medical Sciences, College of Medicine, King Saud bin Abdulaziz University for Health Sciences; b King Abdullah International Medical Research Center, Riyadh, Saudi Arabia.

**Keywords:** anthropometry, forensic anthropology, regression, Saudi, stature, upper limb

## Abstract

Estimating stature based on body/limb parts can help define the characteristics of unidentified bodies. The most studied upper limb part is the hand, although few studies have examined whether stature can be estimated using fingers plus other hand dimensions. Moreover, there is paucity in anthropometric studies that determined whether bilateral whole limb parts (e.g., arms, forearms, and hands) are related to stature among the living subjects.

This prospective cross-sectional study aimed to evaluate the relationship between different upper limb measurements and the stature of Saudi men. Furthermore, I assessed whether upper limb asymmetry was present, and developed regression models to estimate stature based on different available measurements. Stature and 13 upper limb parameters were measured for 100 right-handed Saudi men who were 18 to 24 years old.

All measurements were positively correlated with stature (*P* < .001), and the best single predictor was the bilateral ulnar length. Asymmetry was more pronounced in the hand measurements. A multiparameter model provided reasonable predictive accuracy (±3.77–5.68 cm) and was more accurate than single-parameter models. Inclusion of the right-side fingers improved the model's accuracy.

This study developed potential models for estimating stature during the identification of bodies of Saudi men.

## 1 Introduction

The fundamental goal of any investigation that involves an unidentified human corpse is to establish identification, regardless of the condition of the remains. However, this task is complicated when there are multiple remains (e.g., because of natural disaster, war, mass accidents, or genocide) or when the integrity of the remains has been compromised (e.g., because of mutilation or explosion). In these scenarios, multiple biological parameters must be used to deduce a presumptive identity, which can involve age, stature, and personal characteristics, such as previous dental procedures, trauma, or chronic skeletal disease. Stature allows forensic investigators to match the records of possible victims based on 2 potential strategies. The first is based on the degree of integrity and completeness of skeletal body parts, which can be directly measured when all skeletal components are recovered. A second strategy involves mathematically estimating stature when the skeletal integrity and/or completeness is jeopardised.^[1]^ The mathematical estimation has become increasingly popular, and can be based on different methods, such as multiplication factors or regression models.^[2]^

Stature is mainly controlled by genetic factors, although it is also influenced by environmental, nutritional, socioeconomic, and climate factors,^[3–6]^ which also influence the relationships between stature and variable anatomical measurements.^[7]^ Relative to proximal body parts, distal limb bones or parts are disproportionately influenced by various stressors, especially nutritional and environmental factors, and these changes are more pronounced in men.^[8]^ Furthermore, secular changes are observed in intralimb proportions and stature.^[9]^ Unfortunately, previous reports have indicated that population-specific genetic and environmental factors can lead to large estimation errors when estimation equations for one specific population are applied to a different population.^[10,11]^ Therefore, the best stature estimation models are specific to a single population.

Many studies that have aimed to estimate stature used lower limb bones or body parts, rather than upper limb bones/parts, which was attributed to their direct contribution to stature and higher likelihood of being preserved (vs upper limb components).^[12–14]^ Nevertheless, forensic investigations may need to consider other body parts/bones, such as craniofacial parameters,^[15]^ the vertebrae,^[16]^ and/or the sternum.^[17]^ Although there is near-consensus in the literature that upper limb parts are less accurate than lower limb parts for predicting stature, there is also substantial population-specific variability in the relationships between upper limb parts and stature. Furthermore, studies of living subjects have generally evaluated hand-based parameters, and only a few studies have incorporated long bone lengths and breadths, or the use of multiple upper limb parts to estimate stature. One study estimated stature among Turkish individuals based on upper limb parameters, which included 4 lengths (total arm, upper arm, forearm, and hand) and 2 breadths (wrist and hand), and revealed that forearm length was most effective for estimating the stature of male individuals.^[18]^ A similar study evaluated the lengths of the medial 4 fingers and hand breadth among Iranian individuals, which revealed that hand length was the best parameter for estimating stature of male individuals.^[19]^ Another study of male Iranians revealed that the total upper limb length was better for predicting stature than the hand alone.^[20]^ A study of male Australians also revealed that stature was most accurately estimated using forearm length, rather than hand length, palm length, and hand breadth.^[21]^ Another study of Sudanese Arabs revealed that stature was most accurately predicted using ulnar length, rather than the upper arm or hand lengths with wrist and hand breadths.^[22]^

Limb asymmetry involves morphological differences between paired limb parts, and different populations had varying types and degrees of asymmetry.^[23]^ Furthermore, the upper limb bones exhibit greater asymmetry than the lower limb bones.^[24]^ Although it is tempting to assume that paired limb parts are symmetrical, this assumption is risky and could confound the mathematical estimation of stature in a forensic setting. Sexual differences in asymmetry persist with men exhibiting more pronounced differences in circumference and breadth measurements. Most related studies have assessed adult upper limb asymmetry using direct or radiological evaluation of bones, and few studies have evaluated the hand parameters of living adults. One study of asymmetry in Gujjar Indian adults used 7 measurements to indirectly calculate 6 dimensions (total upper limb, upper arm, forearm, and hand lengths, as well as 2 lower limb parameters), which revealed asymmetry in all variables and recommended side-specific equations even when the asymmetry was not considered significant.^[25]^ However, a study of Sri Lankans revealed no significant asymmetry in the ulnar measurements,^[26]^ and an Australian study revealed very small nonsignificant differences in the mean values for forearm length, hand breadth and length, and palm length.^[21]^ Studies of Sudanese and Iranian individuals have considered multiple body parts but only considered the left side or did not assess asymmetry.^[19,20,22]^

There is limited research regarding biological attributes in the Arabian Peninsula and southwest Asia, and the existing research has mainly focused on the hands. Moreover, few studies have been able to evaluate and compare international results regarding stature estimation based on multiple upper limb parts, such as the radius, ulna, and hand, or related degrees of asymmetry. Therefore, the present study aimed to evaluate whether stature was mathematically related to various upper limb part measurements among Saudi men, as well as whether asymmetry was present in these measurements. This information may be useful for guiding comparative studies of the Arabian population and other international populations, as well as the development of mathematical methods for estimating stature based on different body parts.

## 2 Materials and methods

This study was conducted as prospective cross-sectional study using convenient sampling technique. Data were collected from 100 Saudi men who were students at [name removed for the purpose of the review process], Riyadh, Saudi Arabia. The mean age of these subjects was 20.5 years (range: 18–24 years). In this study this age range was selected because average adult stature is reached by the age of approximately18 years, with an incremental increase until the age of 23.5 years, and then begins decreasing after the age of 25 years with a pronounced decrease after the age of 40 years.^[27–29]^ The study protocol was approved by the institutional review board at King Abdullah International Medical Research Centre, National Guard Health Affairs, Saudi Arabia Saudi Arabia. All subjects provided informed consent before participating in the study.

The subjects completed questionnaires to collect data regarding date of birth, handedness, grandparents’ tribe(s), and medical history. The completed questionnaires were anonymised to ensure that the subjects’ identities were protected. Subjects were considered eligible if they were right-handed, had parents of Arabic ethnicity, and were born and registered as Saudi citizens. Subjects were excluded if they had developmental or physical abnormalities, or if they had pathologies, chronic illnesses, or a surgical history that might have influenced their stature or upper limb dimensions.

Stature and 13 bilateral anthropometric parameters were measured for each participant using standard instruments in a well-lit room (Table 1).^[18,30–32]^ Each measurement was repeated twice by the same observer, and the mean value was recorded to the nearest millimeter. All measurements were conducted at the same time of the day (10 am–3 pm) to avoid confounding from diurnal variations in stature.

**Table 1:** Definition and techniques of the measurements used in the present study.

Abbreviation	Definition and instrument used
S	Straight vertical distance from the vertex and the floor, while subjects are barefooted and erect maintaining the anatomical position and Frankfort plane for the face^∗^
AL	Straight distance between the marked inferior border of the acromion and radiale.^†^
EB	Straight distance between the 2 most projected points of the medial and lateral epicondyles of the humerus. Taken from the back with elbow flexed to 90°.^‡^
UL	Straight distance between olecranon and stylion ulnare while the elbow was flexed to 90° and the fingers were extended in the direction of the long axis of the forearm.^†^
RL	Distance between radiale and stylion.^†^
WB	Distance between the ulnar and radial styloid processes.^‡^
HL	Straight distance between the midpoint of the interstyloid line to the dactylion of the middle finger.^‡^
HB	Straight distance between metacarpal radiale and metacarpal ulnare.^‡^
PL	Straight distance between midpoint of the most distal crease of the wrist and the midpoint of the most proximal flexion crease of the middle finger.^‡^
1DL-5DL	Straight distance between the proximal flexion crease of the finger and dactylion of the same finger.^‡^

1DL-5DL = finger lengths, AL = arm length, EB = elbow breadth, HB = hand breadth, HL = hand length, PL = palm length, RL = radial length, S = stature, UL = ulnar length, WB = wrist breadth.

∗ Seca 217 portable stadiometer (Seca, China).

† Harpenden anthropometer (Holtain Ltd, Crosswell, UK).

‡ Digital sliding caliper (Mitutoyo, Japan).

Before data collection was started, intraobserver error was evaluated by measuring a randomly selected subset of 15 subjects once and then a second time 1 week later. The present study considered measurements acceptable if the relative technical error of measurement was < 5% and if the coefficient of reliability (*R*) was > 0.95.^[33–35]^

The data analyses were performed using IBM SPSS software (version 21.0; IBM Corp, Armonk, NY). The measurement data were evaluated for normality based on skewness and kurtosis, which revealed normal distributions for all variables. Thus, the results were reported as mean, standard deviation, and range. Asymmetry in the left-side and right-side parameters was evaluated using the paired *t* test. Pearson's correlation coefficients were calculated to evaluate whether stature was related to the various upper limb part measurements, and significant correlations were considered present at *P* values of < 0.05. Simple linear regression analyses were applied to the 12 bilateral upper limb measurements. Multiple regression equations were also used to develop models for estimating stature based on the possibility of being able to obtain complete or incomplete measurements (e.g., in forensic settings with only the arm, forearm alone, forearm and hand, hand alone, or hand missing the distal part of the middle finger). The accuracies of the various models were compared using the standard error of the estimate (SEE), with lower SEE values indicating better model accuracy. The model fit and utility of each parameter was evaluated using the *R*^*2*^ coefficient.

## 3 Results

The precision study showed excellent reproducibility for intraobserver errors with *R* ≥0.997 and relative technical error of measurement, < 1.423%.

Table 2 shows the results for stature and the 13 bilateral upper limb measurements for the 100 Saudi men. The mean stature was 172.98 ± 6.16 cm. The arm length measurements exhibited the greatest standard deviation, which was followed by the forearm bones, and the least standard deviation was observed for the bilateral wrist breadth.

**Table 2:** Descriptive statistics for stature and upper limb measurements in centimeters in both sides.

Parameter	Side	Mean	SD	Minimum	Maximum
Stature		172.98	6.16	160.60	191.30
AL	Right	32.24	1.72	28.60	36.00
	Left	32.21	1.74	28.60	36.00
EB	Right	6.75	0.43	5.70	8.00
	Left	6.79	0.42	5.75	7.90
RL	Right	26.40	1.36	23.30	30.60
	Left	26.39	1.40	22.30	30.20
UL	Right	28.40	1.37	24.60	31.70
	Left	28.36	1.40	24.40	31.70
WB	Right	5.26	0.34	4.40	6.10
	Left	5.25	0.34	4.55	6.05
HL	Right	18.67	0.93	16.40	20.70
	Left	18.72	0.93	16.50	20.80
HB	Right	8.19	0.48	7.00	9.45
	Left	8.14	0.47	6.90	9.32
PL	Right	10.77	0.56	9.35	12.10
	Left	10.76	0.54	9.45	12.20
1DL	Right	6.55	0.38	5.60	7.40
	Left	6.58	0.40	5.80	7.50
2DL	Right	7.13	0.40	6.25	8.15
	Left	7.15	0.39	6.10	8.02
3DL	Right	7.97	0.46	6.90	9.07
	Left	7.99	0.46	6.95	8.99
4DL	Right	7.36	0.46	6.30	8.45
	Left	7.36	0.45	6.45	8.30
5DL	Right	6.13	0.43	4.90	7.16
	Left	6.07	0.44	4.90	7.16

1DL-5DL = finger lengths, AL = arm length, EB = elbow breadth, HB = hand breadth, HL = hand length, PL = palm length, RL = radial length, SD = standard deviation, UL = ulnar length, WB = wrist breadth.

Results regarding asymmetry in the upper limb measurements are shown in Table 3. The mean differences between the right and left measurements were small (0.01–0.06 cm), although significant differences were observed in the measurements for elbow breadth, hand length and breadth, and little finger length. The mean values were larger for left-side elbow breadth, hand length, and the 3 lateral finger lengths. However, the mean values were also larger for right-side arm length, forearm bone lengths, wrist breadth, hand breadth, palm length, and the medial 2 finger lengths. The maximum asymmetry was observed for hand breadth followed by the little finger length, whereas the least asymmetry was observed for radius length followed by the ring finger length.

**Table 3:** Bilateral asymmetry in upper limbs measurements.

Variable	Paired *t* test	*P* (two-tailed)
AL	1.17	.244
EB	−2.43	.017
RL	0.12	.908
UL	1.82	.072
WB	1.47	.145
HL	−2.60	.011
HB	3.47	.001
PL	0.40	.688
1DL	−1.65	.102
2DL	−1.61	.111
3DL	−0.90	.370
4DL	0.30	.764
5DL	2.75	.007

1DL-5DL = finger lengths, AL = arm length, EB = elbow breadth, HB = hand breadth, HL = hand length, PL = palm length, RL = radial length, UL = ulnar length, WB = wrist breadth.

Table 4 shows that all 13 upper limb measurements were significantly correlated with stature (*P* < 001). Stature was generally more strongly correlated with length measurements than with breadth measurements, and the strongest correlations were observed with ulnar length (right side: *r* = 0.727, left side: *r* = 0.701) and with arm length (right side: *r* = 0.684, left side: *r* = 0.685). Stature was more strongly correlated with hand breadth (right side: *r* = 0.515, left side: *r* = 0.489) than with elbow breadth or wrist breadth. Among the finger parameters, the greatest correlation was observed with the bilateral index finger length, although right finger measurements were more strongly correlated with stature than left finger measurements. In contrast, left arm measurements were more strongly correlated with stature than right arm measurements.

**Table 4:** Karl Person's correlation between stature and upper limbs measurements.

	Value of r	
Variable	Right	Left
AL	0.684^∗^	0.685^∗^
EB	0.458^∗^	0.471^∗^
RL	0.670^∗^	0.685^∗^
UL	0.727^∗^	0.701^∗^
WB	0.454^∗^	0.400^∗^
HL	0.630^∗^	0.632^∗^
HB	0.515^∗^	0.489^∗^
PL	0.581^∗^	0.548^∗^
1DL	0.566^∗^	0.513^∗^
2DL	0.606^∗^	0.589^∗^
3DL	0.571^∗^	0.567^∗^
4DL	0.577^∗^	0.541^∗^
5DL	0.458^∗^	0.441^∗^

1DL-5DL = finger lengths, AL = arm length, EB = elbow breadth, HB = hand breadth, HL = hand length, PL = palm length, r = correlation coefficient, RL = radial length, UL = ulnar length, WB = wrist breadth.

∗ Significant at 0.001 level (2 tailed).

Table 5 shows the simple linear regression equations for estimating stature based on right-side and left-side measurements. The SEE values are inversely proportional to the accuracy of the stature estimation model. The greatest coefficients of determination were observed for ulnar length (right side: *R*^*2*^ = 0.528, left side: *R*^*2*^ = 0.492), followed by right arm length (*R*^*2*^ = 0.507) and left arm length (*R*^*2*^ = 0.491). The smallest SEE values were observed for right ulnar length (±4.25 cm) and left ulnar length (±4.41 cm), while the highest SEE values were observed for right wrist breadth (±5.52) and left wrist breadth (±5.68). Among the hand measurements, the lowest SEE value was observed for hand length and the highest SEE value was observed for the bilateral little finger measurements.

**Table 5:** Linear regression equations for stature estimation in centimeters using single upper limb measurement.

Parameter	Side	Regression equation	± SEE	*R*^2^	*P* value
AL	Right	S = 94.25 + 2.44 × AL	± 4.52	0.467	<.001
	Left	S = 94.95 + 2.42 × AL	± 4.51	0.469	<.001
EB	Right	S = 128.19 + 6.63 × EB	± 5.51	0.210	<.001
	Left	S = 126.51 + 6.85 × EB	± 5.46	0.222	<.001
RL	Right	S = 92.61 + 3.05 × RL	± 4.60	0.450	<.001
	Left	S = 93.51 + 3.01 × RL	± 4.51	0.469	<.001
UL	Right	S = 80.02 + 3.27 × UL	± 4.25	0.528	<.001
	Left	S = 85.43 + 3.09 × UL	± 4.41	0.492	<.001
WB	Right	S = 129.23 + 8.31 × WB	± 5.52	0.207	<.001
	Left	S = 134.90 + 7.26 × WB	± 5.68	0.160	<.001
HL	Right	S = 95.11 + 4.17 × HL	± 4.81	0.397	<.001
	Left	S = 94.77 + 4.18 × HL	± 4.80	0.399	<.001
HB	Right	S = 118.74 + 6.63 × HB	± 5.31	0.265	<.001
	Left	S = 120.96 + 6.39 × HB	± 5.40	0.239	<.001
PL	Right	S = 103.59 + 6.44 × PL	± 5.04	0.337	<.001
	Left	S = 106.24 + 6.20 × PL	± 5.18	0.300	<.001
1DL	Right	S = 113.55 + 9.07 × 1DL	± 5.11	0.320	<.001
	Left	S = 121.19 + 7.87 × 1DL	± 5.31	0.263	<.001
2DL	Right	S = 106.68 + 9.30 × 2DL	± 4.93	0.367	<.001
	Left	S = 105.99 + 9.37 × 2DL	± 5.01	0.347	<.001
3DL	Right	S = 112.70 + 7.56 × 3DL	± 5.09	0.326	<.001
	Left	S = 112.29 + 7.60 × 3DL	± 5.10	0.321	<.001
4DL	Right	S = 116.03 + 7.73 × 4DL	± 5.06	0.333	<.001
	Left	S = 118.96 + 7.34 × 4DL	± 5.21	0.293	<.001
5DL	Right	S = 132.91 + 6.54 × 5DL	± 5.51	0.209	<.001
	Left	S = 135.79 + 6.12 × 5DL	± 5.56	0.195	<.001

1DL-5DL = finger lengths, AL = arm length, EB = elbow breadth, HB = hand breadth, HL = hand length, PL = palm length, *R*^*2*^ = coefficients of determination, RL = radial length, S = stature, SEE = standard error of estimate, UL = ulnar length, WB = wrist breadth.

Various models were derived based on different assumptions (all parts present, a limb without the hand, or a hand alone) to identify the optimal factors for estimating stature among Saudi men (Table 6). When all parts were present on the right side, the optimal model consisted of right ulnar length, right thumb length, and right arm length. When all parts were present on the left side, the optimal model consisted of left hand length, left ulnar length, and left arm length. When I considered a limb without the hand, the optimal model considered the bilateral ulnar lengths. When I considered a hand alone, the optimal model considered the right hand length and right thumb length or the left hand length alone. The lowest SEE values were observed in the scenario with all parts present on the right side (±3.77 cm) or on the left side (±3.93 cm). Different combinations were used to develop multiple regression equations (Table 7), and right-side measurements consistently provided slightly better estimation than left-side measurements.

**Table 6:** Stepwise regression equations for estimation of stature in centimeters from bilateral upper limb measurements.

Side	Regression equation	± SEE	*R*^2^	*P* value
Right				
Limb	S = 80.02 + 3.27 × UL	± 4.25	0.528	<.001
	S = 66.53 + 2.67 × UL + 4.70 × 1DL	± 3.96	0.596	<.001
	S = 60.91 + 1.72 × UL + 4.40 × 1DL + 1.06 × AL	± 3.77	0.637	<.001
Limb minus hand	S = 80.02 + 3.27 × UL	± 4.25	0.528	<.001
	S = 72.91 + 2.20 × UL + 1.17 × AL	± 4.04	0.578	<.001
Hand only	S = 95.11 + 4.17 × HL	± 4.81	0.397	<.001
	S = 89.34 + 3.01 × HL + 4.19 × 1DL	± 4.68	0.434	<.001
Left				
Limb	S = 85.43 + 3.09 × UL	± 4.41	0.492	<.001
	S = 76.98 + 1.90 × UL + 1.31 × AL	± 4.15	0.556	<.001
	S = 64.87 + 1.17 × UL + 1.22 × AL + 1.90 × HL	± 3.93	0.605	<.001
Limb minus hand	S = 85.43 + 3.09 × UL	± 4.41	0.492	<.001
	S = 76.98 + 1.90 × UL + 1.31 × AL	± 4.15	0.556	<.001
Hand only	S = 94.77 + 4.18 × HL	± 4.80	0.399	<.001

1DL = first finger length, AL = arm length, HL = hand length, *R*^*2*^ = coefficients of determination, S = stature, SEE = standard error of estimate, UL = ulnar length.

**Table 7:** Multiple (direct) regression equations for estimation of stature in centimeters from bilateral upper limb measurements.

Parameters	Side	Regression equation	± SEE	*R*^2^	*P* value
AL, EB	Right	S = 78.86 + 2.13 × AL + 3.75 × EB	± 4.28	0.527	<.001
	Left	S = 83.32 + 2.09 × AL + 3.28 × EB	± 4.35	0.511	<.001
UL, RL, WB	Right	S = 78.40 + 0.48 × RL + 2.73 × UL + 0.86 × WB	± 4.28	0.532	<.001
	Left	S = 82.88 + 1.27 × RL + 1.81 × UL + 0.99 × WB	± 4.39	0.508	<.001
HL, HB	Right	S = 90.61 + 3.39 × HL + 2.34 × HB	± 4.76	0.415	<.001
	Left	S = 91.21 + 3.59 × HL + 1.79 × HB	± 4.78	0.410	<.001
PL, HB	Right	S = 91.02 + 4.76 × PL + 3.75 × HB	± 4.83	0.399	<.001
	Left	S = 93.31 + 4.58 × PL + 3.74 × HB	± 4.97	0.361	<.001
Fingers	Right	S = 98.88 + 4.09 × 1DL + 6.92 × 2DL – 2.48 × 3DL + 4.43 × 4DL – 2.41 × 5DL	± 4.80	0.424	<.001
	Left	S = 101.42 + 2.18 × 1DL + 6.02 × 2DL + 2.24 × 3DL + 1.00 × 4DL – 1.82 × 5DL	± 5.01	0.372	<.001

1DL-5DL = finger lengths, AL = arm length, EB = elbow breadth, HB = hand breadth, HL = hand length, PL = palm length, *R*^*2*^ = coefficients of determination, RL = radial length, S = stature, SEE = standard error of estimate, UL = ulnar length, WB = wrist breadth.

## 4 Discussion

Anthropometry facilitates a quantitative evaluation of the human body and skeleton, as well as comparisons of stature and intralimb or inter-limb proportions.^[7,36,37]^ This technique is widely accepted for forensic applications, as it is cost-effective and noninvasive.^[38]^ Nevertheless, its application requires standardized methods and well-defined landmarks to ensure that the data are reliable and reproducible, especially for the mathematical determination of body part parameters.^[28]^ The present study aimed to address these issues by using well-defined anatomical landmarks, accurate instruments, standardized measurement techniques, confirmation of precision and reliability before the data collection, and an assessment of asymmetry.

The results revealed that bones in the right arm/forearm had marginally longer lengths than bones in the left arm/forearm, although larger values were observed for left elbow breadth (vs right elbow breadth) and right wrist breadth (vs left wrist breadth). Previous studies had also indicated that right upper limb parts are typically 1% to 3% longer than left upper limb parts,^[39]^ while hand breadth values are larger on the right side. Left fingers were generally longer, although the right little finger was longer than its left counterpart, and similar lengths were observed for the ring fingers on both sides. These findings are in agreement with previous reports which demonstrated that right-handed subjects exhibit stronger right-directional linear growth patterns in proximal bones and that hand breadth is largely governed by hand preference.^[23,40]^ Left fingers were generally longer, although the right little finger was longer than its left counterpart, and similar lengths were observed for the ring fingers on both sides. This finding concurs with a previous report which showed that men tend to have longer left fingers than right fingers.^[41]^ Differences in upper limb measurements were compared between Saudi men and other populations via a meta-analysis, using Cohen's d method to quantify the effect sizes between Saudi men and the other populations (Table 8). The differences were classified based on the Cohen's d value as very small (0.01), small (0.2), medium (0.5), large (0.8), very large (1.2), and huge (2.0).^[42]^ Relatively small differences in stature were observed between Saudi men and Egyptians (0.03) or a mixed Turkish population (0.08),^[43,44]^ although a huge difference was observed between Saudi men and northeastern Indians (2.05).^[45]^ A comparison of left arm lengths revealed a medium difference between Saudi men and Sudanese individuals (0.32),^[22]^ but a huge difference between Saudi men and Iranians (2.07).^[19]^ Elbow breadth could not be compared because no previous studies have aimed to estimate stature based on elbow breadth. A medium difference in left ulnar length was observed between Saudi men and Sudanese individuals.^[22]^ A huge difference was observed in the left radius length between Saudi men and Iranians (3.08),^[19]^ while only a medium difference in bilateral radius length was observed between Saudi men and Australians.^[21]^ A small difference in hand length was observed between Saudi men and Slovak individuals (right side: 0.03, left side: 0.011)^[46]^ or Malaysians individuals (right side: 0.17, left side: 2.05),^[47]^ although substantial bilateral difference was observed between Saudi men and northern Indians (right side: 1.36, left side: 1.25)^[31]^ or a group of Egyptians (right side: 1.34, left side: 1.39).^[43]^ The largest difference in hand breadth was observed between Saudi men and Australians (right side: 1.90, left side: 1.87),^[48]^ and the smallest difference was observed for the left side between Saudi men and Egyptians (0.02),^[43]^ and for the right side between Saudi men and Chinese Han individuals (0.02).^[49]^ Large differences in palm length were observed between Saudi men and Australians^[21,48]^ or Koreans.^[50]^ Most of the finger measurements exhibited medium to very large differences between Saudi men and the other populations. However, a small difference was observed in the right ring fingers between Saudi men and Koreans (0.11).^[50]^ The greatest overall difference in the left finger measurements was generally between Saudi men and Iranians, although an exception was the little finger for Malaysians.^[19,47]^ These population-specific differences in stature and upper limb dimensions are related to genetic and ethnic factors, as well as differences in environmental factors, nutritional factors, and levels of physical activity.^[51]^

**Table 8:** Comparison of Saudi males with previously published studies using Cohen's D.

	Egyptian	Egyptian	Sudanese	Iranian	Indian (Northern)	Indian (Southern)	Indian Rajput	Indian	Mauritian (Indo)	Malaysians (iban)	Chinese Han	Korean	Turks	Turks	Slovaks	Australians Western	Australian
	Abdel-Malek ^[43]^(1990)	Habib ^[54]^(2010)	Ahmed ^[22]^(2013)	Akhlaghi ^[19]^(2012)	Rastogi ^[31]^(2008)	Rastogi ^[31]^(2018)	Krishan ^[55]^(2007)	Sen ^[45]^(2014)	Agnihotri ^[53]^(2009)	Zulkifly ^[47]^(2018)	Zhang ^[49]^(2017)	Kim ^[50]^(2019)	Ozaslan ^[18]^(2006)	Ozaslan ^[44]^(2012)	Uhrova ^[46]^(2015)	Ishak ^[48]^ (2012)	Howley ^[21]^(2018)
Stature		0.03	0.24	0.34	0.50	0.22	0.16	0.75	2.05	0.16	1.85	0.45	0.58	0.17	0.08	1.03	0.83
AL																	
Right																	
Left			0.32	2.07									0.93				
EB																	
Right																	
Left																	
UL																	
Right																	
Left			0.61														
RL																	
Right																	0.34
Left				3.08									1.30				0.30
WB																	
Right												2.71		1.30			
Left			1.03										1.31				
HL																	
Right	1.34	0.70			1.36	1.30			0.24	0.17	0.33	0.42		0.60	0.03	0.94	0.99
Left	1.39	0.71	0.43	0.18	1.26	1.18	0.57		0.20	0.19	0.40		0.57		0.01	0.91	0.97
HB																	
Right	0.23				0.33	0.18			0.59	0.69	0.20	0.87		0.23	0.64	1.90	1.72
Left	0.02		0.45	0.95	0.42	0.25	0.09		0.64	0.88	0.26		0.29		0.80	1.87	1.60
PL																	
Right												0.55				0.82	1.01
Left																0.88	0.94
1DL																	
Right										1.13		1.09				0.47	
Left										0.88						0.37	
2DL																	
Right								0.72		0.53		0.24				0.95	
Left				2.48				0.73		0.46						0.94	
3DL																	
Right										0.78		0.30				0.76	
Left				1.24						0.75						0.75	
4DL																	
Right								0.69		0.58		0.11				0.86	
Left				1.37				0.49		0.46						0.96	
5DL																	
Right										0.97		0.55					
Left				1.44						2.21							

1DL-5DL = finger lengths, AL = arm length, EB = elbow breadth, HB = hand Breadth, HL = hand length, PL = palm length, RL = radial length, UL = ulnar length, WB = wrist breadth.

In the present study, the greatest asymmetry was observed in hand breadth (*t* = 3.47, *P* = .001), followed by the little finger length (*t* = 2.75, *P* = 0.007), hand length (*t* = 2.6, *P* = 0.011), and elbow breadth (*t* = 2.60, *P* = .017). Symmetry was generally observed for the other part measurements. It is interesting that only elbow breadth exhibited asymmetry, while the other arm/forearm parts were symmetrical, which suggests that these parameters are not directly related.^[52]^ Furthermore, previous studies of ulnar length revealed symmetry in Indo-Mauritian and Sri Lankan populations.^[26,53]^ Among Saudi men, right elbow breadth was larger, which agrees with the right-handedness of the participants, although a previous study revealed a significantly higher value for left elbow breadth, which suggests that elbow breadth may not be strongly related to handedness.^[52]^ Previous studies regarding asymmetry in hand dimensions have revealed inconclusive results. For example, the asymmetry in hand length among Saudi men agrees with the asymmetrical results among northern Indians (*P* = 0.01) and southern Indians (*P* = 0.002),^[31]^ as well as among Egyptian men (*t* = 2.41, *P* = 0.018), but contradict the lack of significant asymmetry among northern Egyptians.^[43,54]^ The asymmetry I observed among Saudi men also conflicts with the lack of asymmetry that has been observed among Slovaks,^[46]^ Australians,^[48]^ Malaysians,^[47]^ and Rajput Indians.^[55]^ My findings regarding bilateral hand breadth differences agree with other findings among Australians,^[48]^ northern and southern Indians,^[31]^ and Rajput Indians, who had significantly broader right hands.^[55]^ Nevertheless, no significant differences in hand length and breadth were observed in studies of Iban Malaysians,^[47]^ Chinese individuals,^[49]^ and Slovaks.^[46]^ Saudi men had generally symmetrical finger lengths, with the exception of the little finger, which conflicts with reported asymmetry in the thumb and ring fingers of Iban Malaysians and in the ring fingers of northeastern Indians.^[45,47]^ Genetic factors are the main determinants of linear growth and the presence of perfect symmetry indicates ideal development.^[40]^ The current study only included right-handed individuals as established literature indicates that 90% of humans express right-hand dominance in writing and 72% to 96% for various motor skills.^[56,57]^ Therefore, the asymmetry in length and breadth measurements that I observed may be related to differences in physical activity, differential mechanical loading and directional growth related to right-handedness, nutritional factors, environmental factors, latitude, hormones, or stresses during development.^[23,40,51]^

The present study revealed that all upper limb dimensions were positively correlated with stature (*P* < .001), which agrees with previous reports that upper limb dimensions are highly correlated with stature and can be used to estimate stature.^[18,21,22]^ However, I observed different correlations for each side (right side: *r* = 0.458–0.727, left side: *r* = 0.400–0.701), with the strongest correlations observed for the bilateral ulnar measurements (right side: *r* = 0.727, left side: *r* = 0.701) and arm length (right side: *r* = 0.684, left side: *r* = 0.685). Among the hand-based parameters, the strongest correlations with stature were observed for hand length (right side: *r* = 0.630, left side: *r* = 0.632) and index finger length (right side: *r* = 0.606, left side: *r* = 0.589). Interestingly, the correlations between left arm length and stature among Saudi men were stronger than those among Turkish individuals^[18]^ and Iranians,^[19]^ but lower than that among Sudanese individuals.^[22]^ The left ulnar and wrist values were also lower among Saudi men than among Sudanese individuals,^[22]^ although the radius and wrist values were more strongly correlated with stature among Saudi men than among Turkish or Iranian individuals.^[18,19]^ The correlation between hand length and stature was similar among Saudi men and upper Egyptians^[43]^ or Slovaks,^[46]^ but greater than among Turkish and Sudanese individuals,^[18,22]^ and lower than among Iranians,^[19]^ non-Rajput Indians,^[31,55]^ Egyptians,^[54]^ Malaysians,^[47]^ and Australians.^[21,48]^ In contrast, hand breadth was more strongly correlated with stature among Saudi men than among Turkish individuals,^[18]^ Iranians,^[19]^ upper Egyptians,^[43]^ and Slovaks^[46]^ but were similar to those among Malaysians,^[47]^ north Indians,^[31]^ Australians,^[48]^ and Sudanese individuals.^[22]^ The correlation between hand breadth and stature was weaker among Saudi men than among south Indians,^[31]^ and the correlation between palm values and stature was also weaker among Saudi men than among Australians.^[48]^ Among Saudi men, right finger values were generally more strongly correlated with stature than left finger values, and were generally more strongly correlated than among Malaysians individuals, with the exception of the left little finger.^[47]^ In contrast, Saudi men had weaker correlations between stature and the values for left index, middle, and little figures, relative to among Iranians, although Saudi ring fingers were more strongly correlated with stature than Iranian ring findings.^[19]^ These results suggest that ulnar measurements are a better predictor of stature than other upper limb measurements, although hand length is the preferred measurement if only the hand is available. Although most of the reported studies used right-handed subjects as their inclusion criteria, the remaining studies that included all subjects irrespective of hand dominance reported that more than 90% of their sample were right handed.^[21]^ Stature, body proportions, and hand preference are mainly governed by genetics, hormones, and environmental factors. Previous reports showed that right-handed subjects are taller than left-handed.^[58]^ Moreover, the right-hand dominance can cause increased right directional linear growth and muscle mass reflecting in final expression of size and shape which in turn affects stature correlation with upper limb parts.^[25,59]^ Moreover, the differential human responses to stresses during development can result in small random deviations from this general pattern.^[60]^ There are clear population-specific variations in the relationships between limb/part measurements and stature, which are related to diverse factors, including nutrition, physical activity, environment, and genetic factors. Therefore, population-specific models should be used when attempting to determine stature based on body part measurements.

In the present study, the SEE values for predicting stature based on different upper limb dimensions were ± 4.25 to 5.68 cm among Saudi men. Among these dimensions, the dimensions that were best suited for developing a predictive model (i.e., *r* > 0.69) were bilateral ulnar length (right side SEE: ± 4.25 cm, left side SEE: ± 4.41 cm) and left arm length (SEE: ± 4.51 cm).^[47]^ Some previous studies have indicated that proximal upper limb parts provide better accuracy than distal parts,^[19,61]^ although my finding was that distal parts provide better prediction, and this agrees with previous findings among Turkish and Sudanese Arab populations.^[18,22]^ These differences might be explained by ethnicity-related differences, as the relationship between ulnar length and stature is influenced by ethnicity.^[61]^ Moderate correlations were observed between stature and the right arm and index finger lengths, as well as the bilateral radial and hand lengths (r = 0.60–0.68). Thus, among Saudi men, ulnar length is superior to the radius length for estimating stature. Osteological and radiological studies have revealed inconsistent findings, as results among German individuals agree with my findings, while the radius was preferred over the ulna in studies of Turkish, northern Thai, and Japanese individuals.^[62–65]^ Methodological and/or genetic differences may explain the inconsistency in those findings. In the same upper limb part, breadth values had less accuracy (SEE: ± 5.31 cm or more) than length values among Saudi men, which agree with the findings from studies that evaluated individuals from Egypt,^[43,54]^ Sudan,^[22]^ Malaysia,^[47]^ India,^[31,55]^ Korea,^[50]^ Turkey,^[18]^ Australia,^[21,48]^ and Slovakia.^[46]^ Interestingly, I am not aware of any studies regarding using elbow breadth to estimate stature. Table 9 compares the various SEE values from among Saudi men and other populations, which revealed that the SEE value for a model that incorporated ulnar length, arm length, and wrist breadth in Saudi men was higher than the SEE value for the same model among Sudanese individuals,^[22]^ but lower than the SEE value for a model that incorporated arm length and wrist breadth among Turkish individuals.^[18]^ The SEE value for radius length was lower among Saudi men than among Australians,^[21]^ but higher than the value among Turkish individuals.^[18]^ Among the various hand parameters, hand length consistently provided the best SEE values, which were lower among Saudi men than among individuals from Egypt,^[43,54]^ Sudan,^[22]^ Malaysia,^[47]^ India,^[31,55]^ Korea,^[50]^ Turkey,^[18]^ and Slovakia.^[46]^ However, the SEE value among Saudi men was greater than the value among Australians.^[21]^ Moreover, Saudi men tended to have lower values for hand breadth and palm breadth, relatively to western Australians, albeit with comparable hand length values.^[48]^ Among Saudi men, the accuracy of stature prediction based on hand breadth exceeded that of wrist or elbow breadth.

**Table 9:** Comparison of standard error of estimation in different male population studies.

		Saudis	Egyptian	Sudanese Arabs	Malaysia (Iban)	Indian (Rajput)	Indian (Northern)	Indian (Southern)	Korean	Turks	Australian	Australian (Western)	Slovak
Variable	Side	Present	Habib ^[54]^(2010)	Ahmed ^[22]^(2013)	Zulkifly ^[45]^(2018)	Krishan^[55]^(2007)	Rastogi ^[31]^(2008)	Rastogi ^[31]^(2008)	Kim ^[50]^(2019)	Ozaslan ^[18]^(2006)	Howley ^[21]^(2018)	Ishak ^[48]^(2012)	Uhrova^[44]^(2015)
AL	Right	± 4.52											
	Left	± 4.51		± 4.48						± 5.94			
EB	Right	± 5.51											
	Left	± 5.46											
UL	Right	± 4.60											
	Left	± 4.51		± 4.31									
RL	Right	± 4.25									± 3.89		
	Left	± 4.41								± 5.25	± 3.94		
WB	Right	± 5.52											
	Left	± 5.68		± 5.34						± 6.53			
HL	Right	± 4.81	± 5.30		± 4.97	± 5.22	± 4.99	± 4.83	± 5.09		± 4.46	± 4.83	± 5.01
	Left	± 4.80	± 5.48	± 5.01	± 5.30	± 5.17	± 4.96	± 4.94		± 5.62	± 4.26	± 4.74	± 5.03
HB	Right	± 5.31			± 6.64	± 5.60	± 5.74	± 5.71	± 6.04		± 5.05	± 5.96	± 5.96
	Left	± 5.40		± 5.85	± 6.13	± 5.50	± 5.76	± 5.73		± 6.58	± 4.72	± 6.11	± 6.11
PL	Right	± 5.04							± 5.24		± 4.93	± 5.32	
	Left	± 5.18									± 5.05	± 5.27	
1DL	Right	± 5.11			± 6.53				± 6.08				
	Left	± 5.31			± 6.86								
2DL	Right	± 4.93			± 6.23				± 5.67				
	Left	± 5.01			± 6.44								
3DL	Right	± 5.09			± 6.52				± 5.50				
	Left	± 5.10			± 6.23								
4DL	Right	± 5.06			± 6.46				± 5.71				
	Left	± 5.21			± 6.46								
5DL	Right	± 5.51			± 7.11				± 6.05				
	Left	± 5.56			± 6.55								

1DL-5DL = finger lengths, AL = arm length, EB = elbow breadth, HB = hand breadth, HL = hand length, PL = palm length, RL = radial length, UL = ulnar length, WB = wrist breadth.

A stepwise method was used to develop a multifactor model that provided lower SEE values than single-factor models, although I observed side-specific differences in the relevant measurements. For example, the model based on right limb parameters incorporated ulnar length, thumb length, and arm length, while the model based on left limb parameters incorporated ulnar length, arm length, and hand length. Furthermore, the model based on right-limb parameters provided better accuracy than the model based on left-limb parameters in terms of the SEE values (±3.77 cm vs ± 3.93 cm) and the *R*^*2*^ values (0.637 vs 0.605). When I considered the left arm/forearm parameters, the left ulnar length was considered the preferred left-side predictor of stature, while the preferred right-side predictors were arm length and ulnar length. Among the hand parameters, the preferred right-side predictors were hand length and thumb length, while the preferred left-side predictor was hand length. A previous study of Australians used 4 upper limb dimensions and identified 2 right-side predictors but only 1 left-side predictor.^[21]^ Few studies have evaluated bilateral hand measurements, including fingers, for estimating stature in different populations, which makes comparisons difficult. Nevertheless, my findings agree with the use of 1 finger length to improve estimations based on right-side measurements among Malaysian and western Australian individuals.^[47,48]^ These findings are important in forensic practise, as most studies of living subjects have evaluated left-side upper limb long bones based on the assumption of symmetrical relationships, although this is not true in all populations, and population-specific models are needed to consider which limb(s) and parameter(s) are preferable. The hand has received the greatest amount of attention for estimating stature, and measurements are generally performed bilaterally, although very few studies have incorporated the fingers. Therefore, my findings, and those among Malaysian and Australian individuals, indicate that it may be important to incorporate finger measurements when using hand parameters to estimate stature. My results also suggest that using various body parts from different scenarios can be combined into models that provide lower SEE values than single-parameter models, and similar results have been reported in other populations. Thus, when forensic investigators are confronted with the upper limb(s) of an unidentified Saudi man, a customized model for predicting stature might be selected based on the available parameters from the limb(s).

The present study has limitations that should be considered. First, I only considered male subjects, as there are local regulations that restrict male examiners from performing the measurements for female subjects for research purposes. Thus, studies are needed to develop similar estimation models for Saudi women. Second, although most subjects lived in the Saudi capital, they were originally from many different locations within Saudi Arabia. Additional studies in the Arabian Peninsula and of Asian Arabs are recommended to evaluate regional differences in intralimb and interlimb proportions, the abilities of various body part measurements to predict stature and levels of asymmetry.

## 5 Conclusions

The present study developed various models that can be used to estimate stature among Saudi men using various upper limb dimensions. The most reliable predictors were the bilateral ulnar lengths and the left arm length. Significant asymmetry was observed in the upper limb parameters, especially in the hand parameters, and estimates based on right-side parts were improved by the inclusion of finger dimensions. These results may be useful for application in forensic investigations that aim to predict the statures of Saudi men when dealing with intentional or accidental dismemberment, and a similar study is recommended to develop models for Saudi women. These results may also provide baseline data for further forensic and anthropological studies of Arabic individuals or individuals with mixed Arabic heritage and/or from nearby populations.

## Acknowledgments

This work was conducted at College of Medicine, King Saud bin Abdulaziz University for Health Sciences, Riyadh, Kingdom of Saudi Arabia. Moreover, the author thanks Dr Emad Masuadi for reviewing the statistical analysis of this manuscript.

## Author contributions

**Conceptualization:** Altayeb Abdalla Ahmed.

**Data curation:** Altayeb Abdalla Ahmed.

**Formal analysis:** Altayeb Abdalla Ahmed.

**Funding acquisition:** Altayeb Abdalla Ahmed.

**Investigation:** Altayeb Abdalla Ahmed.

**Methodology:** Altayeb Abdalla Ahmed.

**Project administration:** Altayeb Abdalla Ahmed.

**Validation:** Altayeb Abdalla Ahmed.

**Writing – original draft:** Altayeb Abdalla Ahmed.

**Writing – review & editing:** Altayeb Abdalla Ahmed.
